# Scopoletin Protects against Methylglyoxal-Induced Hyperglycemia and Insulin Resistance Mediated by Suppression of Advanced Glycation Endproducts (AGEs) Generation and Anti-Glycation

**DOI:** 10.3390/molecules20022786

**Published:** 2015-02-09

**Authors:** Wen-Chang Chang, Shinn-Chih Wu, Kun-Di Xu, Bo-Chieh Liao, Jia-Feng Wu, An-Sheng Cheng

**Affiliations:** 1 Department of Medicinal Plant Development, Yupintang Traditional Chinese Medicine Foundation, 4F., No.2, Ln. 138, Yongyuan Rd., Yonghe Dist., New Taipei City 234, Taiwan; E-Mails: d99641001@ntu.edu.tw (W.-C.W.); kuendshe@gmail.com (K.-D.X.); bsnwjay@hotmail.com (B.-C.L.); Kone52074@hotmail.com (J.-F.W.); 2 Department of Animal Science and Technology, National Taiwan University, 59 Roosevelt Road Section 4, Taipei 10617, Taiwan; E-Mail: scw01@ntu.edu.tw

**Keywords:** scopoletin (SP), methylglyoxal (MG), advanced glycation endproducts (AGEs), nuclear factor-erythroid 2-related factor 2 (Nrf2), anti-glycation

## Abstract

Recently, several types of foods and drinks, including coffee, cream, and cake, have been found to result in high methylglyoxal (MG) levels in the plasma, thus causing both nutritional and health concerns. MG can be metabolized by phase-II enzymes in liver through the positive regulation of nuclear factor-erythroid 2-related factor 2 (Nrf2). In this study, we investigated the ability of scopoletin (SP) to protect against MG-induced hyperglycemia and insulin resistance. Recently, SP was shown to be a peroxisome proliferator-activated receptor-γ activator to elevate insulin sensitivity. We investigated the effects of oral administration of SP on the metabolic, biochemical, and molecular abnormalities characteristic of type 2 diabetes in MG-treated Wistar rats to understand the potential mechanism of scopoletin for diabetes protection. Our results suggested that SP activated Nrf2 by Ser40 phosphorylation, resulting in the metabolism of MG into d-lactic acid and the inhibition of AGEs generation, which reduced the accumulation of AGEs in the livers of MG-induced rats. In this manner, SP improved the results of the oral glucose tolerance test and dyslipidemia. Moreover, SP also increased the plasma translocation of glucose transporter-2 and promoted Akt phosphorylation caused by insulin treatment in MG-treated FL83B hepatocytes. In contrast, SP effectively suppressed protein tyrosine phosphatase 1B (PTP1B) expression, thereby alleviating insulin resistance. These findings suggest that SP acts as an anti-glycation and anti-diabetic agent, and thus has therapeutic potential for the prevention of diabetes.

## 1. Introduction

The excessive accumulation of advanced glycation end products (AGEs) contributes related to the development of diabetes [1]. Numerous studies have indicated that carbonyl precursors of AGEs are glycotoxic mediators of carbonyl stress that is related to diabetes, including methylglyoxal (MG), glyoxal, and carboxymethyllysine [2,3]. MG also acts as a molecular species to cause glycative modification of proteins and nucleic acids [4,5]. In clinical experiments, accumulation of MG induces marked cytotoxicity, apoptosis, and inflammatory response *in vitro* and *in vivo* [6,7], and MG levels were found to be elevated in the cerebrospinal fluid of diabetic individuals [8]. The detoxification of MG is mediated by the antioxidation-glyoxalase system occurred in the cytosol of cells [9]. S-d-lactoylglutathione was involved in the conversion of MG to d-lactate in the glyoxalase system [10].

Previous studies have shown that coffee, cream, and cake, have been confirmed to elevate MG levels in serum; coffee contained 230 μM of MG [11], and approximately 5 mg/g MG was contained in honey [12]. Moreover, wine and beer contained 1.6 and 1.0 μg/mL MG, respectively [13,14]. In Spain, people intake 216 μg of MG in cookies per day [15]. According to the abovementioned data, MG intake is an important issue for the population.

Aminoguanidine (AG) has been used as an anti-glycation agent to suppress AGEs production [16]. Coumarins have been shown to exert anti-diabetic and anti-inflammatory activites [17], moreover, coumarins (like 7-hydroxy-6-methoxycoumarin, scopoletin) from *Artemisia capillaris* have also been found to inhibit the generation of AGEs [18]. The focus of this study is scopoletin, a coumarin from *Helianthus annuus* [19], as several chemical and pharmacological studies have demonstrated that scopoletin from other plants such as *Morinda citrifolia,* *Tetrapleura tetraptera* and *Polygala sabulosa* exhibits antimicrobial, anti-inflammatory, antioxidative [20], antihypertensive [21], anti-depressant [22], antitumor [23], and antiarthritic activities [24]. The aim of this study was to investigate the effects of scopoletin on antiglycation and insulin signaling in rats with MG-induced diabetes.

## 2. Results and Discussion

### 2.1. Inhibition of AGEs Production by Scopoletin (SP)

**Figure 1:**
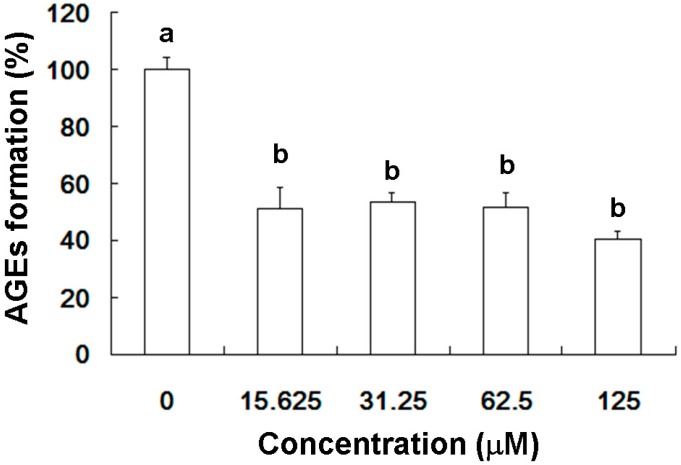
Inhibition of AGEs production by scopoletin (SP). Data here shown as mean ± SD (*n* = 3). (a, b) indicate statistically significant differences *p* < 0.05.

As shown in Figure 1, SP at concentrations of 15.625 to 125 μM SP significantly attenuated AGEs generation. This result indicated that scopoletin has the potential to inhibit glycation.

### 2.2. Improvements of Scopoletin (SP) on Blood Glucose

**Figure 2:**
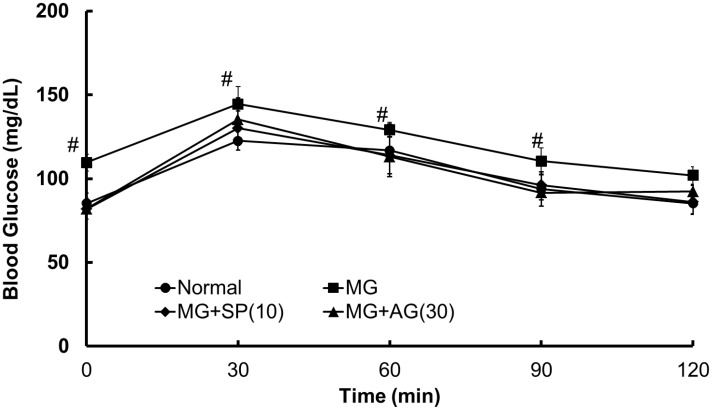
The effects of scopoletin (SP) and aminoguanidine (AG) on OGTT at week-12. Data were shown as mean ± SD (*n* = 8/group). ^#^ The significant difference compared to the normal group (*p* < 0.05). Normal: deionized water; MG: methylglyoxal (300 mg/kg body weight); MG + SP (10): methylglyoxal (300 mg/kg body weight) + scopoletin (10 mg/kg body weight); MG + AG (30): methylglyoxal (300 mg/kg body weight) + aminoguanidine (30 mg/kg body weight).

The levels of fasting blood glucose in MG-treated rats were assessed by the OGTT (glucose, 2 g/kg) on week 12 after overnight fasting (Figure 2).

**Figure 3:**
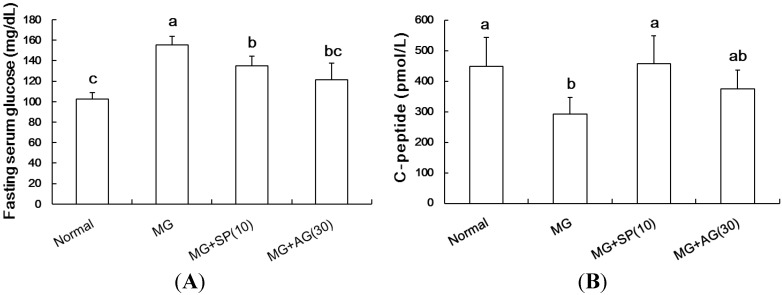
The effects of scopoletin (SP) and aminoguanidine (AG) on (**A**) fasting serum glucose and (**B**) C-peptide. Data were shown as mean ± SD (*n* = 8). (a–c) indicate statistically significant differences *p* < 0.05. Normal: deionized water; MG: methylglyoxal (300 mg/kg body weight); MG + SP (10): methylglyoxal (300 mg/kg body weight) + scopoletin (10 mg/kg body weight); MG + AG (30): methylglyoxal (300 mg/kg body weight) + aminoguanidine (30 mg/kg body weight).

MG treatment resulted in hyperglycemia after 30 min of oral glucose administration, as compared to the control group. However, SP (10 mg/kg, oral administration) and AG (30 mg/kg, oral administration) both attenuated increases in blood glucose in the OGTT. Similarly, the elevation of blood glucose caused by MG administration was alleviated by treatment with SP or AG (Figure 3A) MG treatment markedly reduced serum C-peptide levels. However, SP and AG treatment restored C-peptide serum levels in MG-treated rats (Figure 3B).

### 2.3. Effects of Scopoletin (SP) on Serum Total Cholesterol (TC), Triacylglycerol (TG), HDL-C, LDL-C, and Free Fatty Acid Levels

After MG treatment for 12 weeks, we observed significantly elevated serum TC, LDL-C, and cardiovascular risk index. In contrast, serum HDL-C was decreased in MG-treated rats. However, oral administration of SP or AG effectively mitigated these effects of MG (Table 1).

**Table 1:** Serum TC, LDL-C, and cardiovascular risk index. (a–c) indicate statistically significant differences *p* < 0.05. Data are presented as mean ± SD (*n* = 8/group).

Items/Groups	Normal	MG	MG + SP (10)	MG + AG (30)
Triglyceride (mg/dL)	50.30 ± 1.63 ^a^	52.30 ± 5.99 ^a^	42.30 ± 6.47 ^b^	39.80 ± 7.60 ^b^
Cholesterol-T (mg/dL)	63.00 ± 7.07 ^ab^	72.80 ± 6.15 ^a^	56.50 ± 3.83 ^b^	51.80 ± 6.70 ^b^
Free fatty acid(mmol/L)	1.09 ± 0.08 ^a^	1.11 ± 0.12 ^a^	0.68 ± 0.06 ^b^	0.61 ± 0.10 ^b^
LDL-C (mg/dL)	7.83 ± 0.75 ^b^	11.80 ± 2.56 ^a^	6.80 ± 1.10 ^b^	7.17 ± 0.75 ^b^
HDL-C (mg/dL)	61.90 ± 2.66 ^a^	53.40 ± 4.41 ^b^	61.10 ± 7.13 ^ab^	58.20 ± 8.91 ^ab^
Cardiovascular risk index	1.12 ± 0.04 ^b^	1.25 ± 0.08 ^a^	1.00 ± 0.08 ^c^	1.00 ± 0.07 ^c^

### 2.4. MG Metabolism

**Figure 4:**
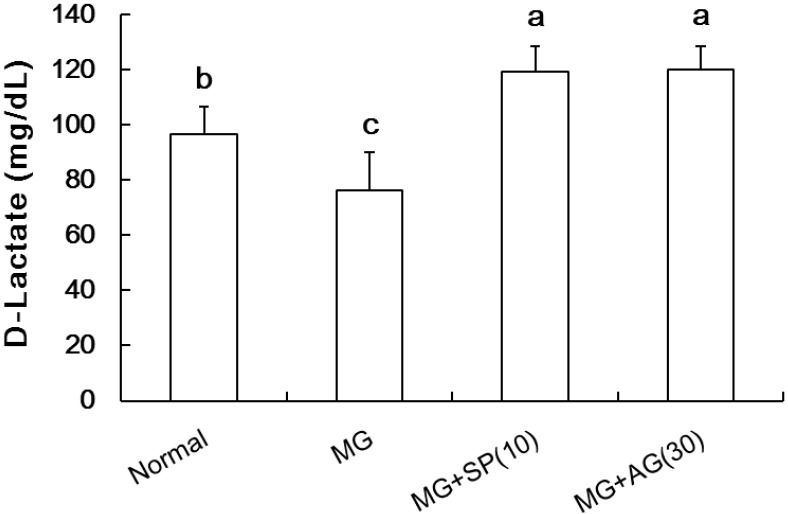
The effects of scopoletin (SP) on serum methylglyoxal (MG) metabolism into d-lactic acid. (a–c) indicate statistically significant differences *p* < 0.05. Data are presented as mean ± SD (*n* = 8/group). Normal: deionized water; MG: methylglyoxal (300 mg/kg body weight); MG + SP (10): methylglyoxal (300 mg/kg body weight) + scopoletin (10 mg/kg body weight); MG + AG (30): methylglyoxal (300 mg/kg body weight) + aminoguanidine (30 mg/kg body weight).

MG could be converted into d-lactic acid mediated by glyoxalase catalysis thereby limiting AGEs generation [25]. The level of d-lactic acid in the serum of MG-treated rats was lower than that of the control group (Figure 4).

**Figure 5:**
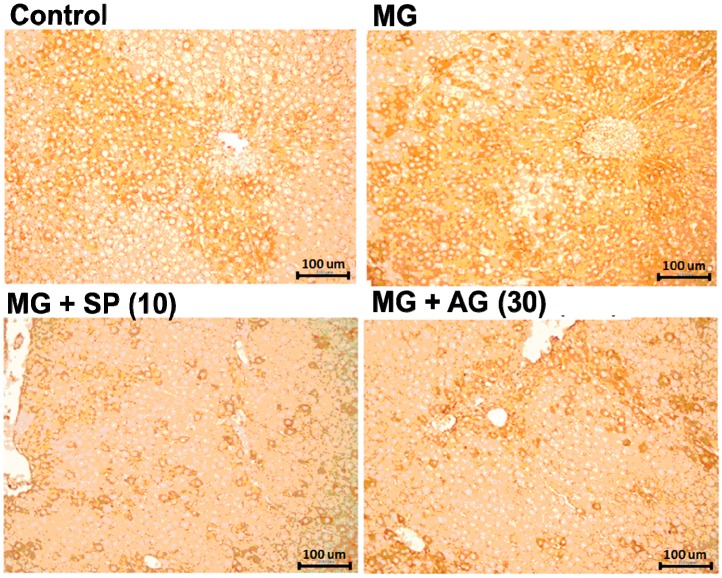

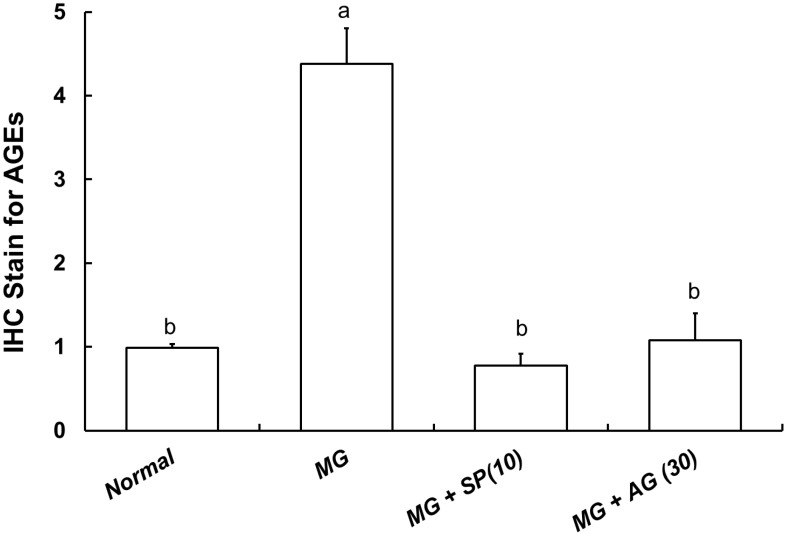
The IHC stain for hepatic AGEs levels. Normal: deionized water; MG: methylglyoxal (300 mg/kg body weight); MG + SP (10): methylglyoxal (300 mg/kg body weight) + scopoletin (10 mg/kg body weight); MG + AG (30): methylglyoxal (300 mg/kg body weight) + aminoguanidine (30 mg/kg body weight). (a,b) indicate statistically significant differences *p* < 0.05. Data are presented as mean ± SD (*n* = 8/group). Bar: 100 micrometer.

In addition, SP treatment significantly elevated d-lactic acid levels in MG-treated rats, suggesting that SP effectively promoted MG metabolism. Furthermore, the accumulation of AGEs in the livers of MG-treated rats was also alleviated by SP and AG treatment, as measured by IHC (Figure 5).

**Figure 6:**
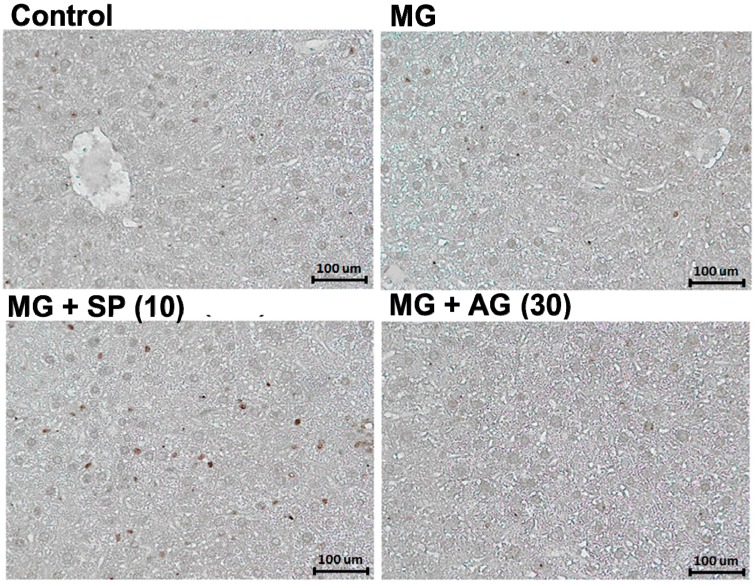

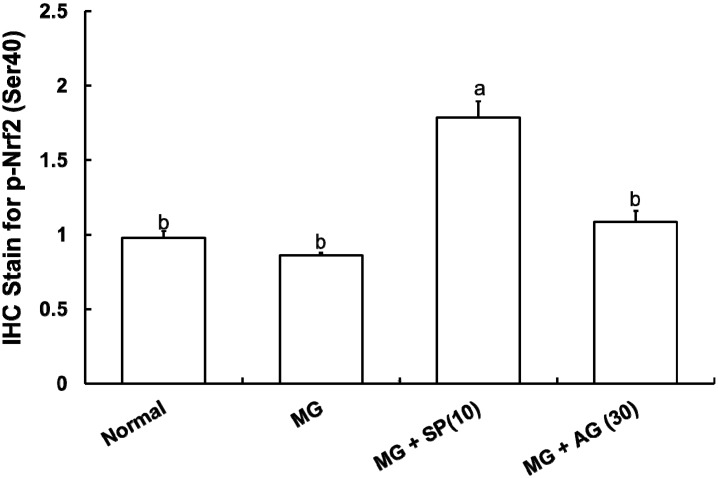
The IHC stain for hepatic p-Nrf2 levels. Normal: deionized water; MG: methylglyoxal (300 mg/kg body weight); MG + SP (10): methylglyoxal (300 mg/kg body weight) + scopoletin (10 mg/kg body weight); MG + AG (30): methylglyoxal (300 mg/kg body weight) + aminoguanidine (30 mg/kg body weight). (a,b) indicate statistically significant differences *p* < 0.05. Data are presented as mean ± SD (*n* = 8/group). Bar: 100 micrometer.

Nrf2 is a transcription factor for antioxidant enzyme [26,27] and glyoxalase expression [25]. Various potential kinase pathways leading to Nrf2 activation (ser40 phosphorylation), including protein kinase C (PKC), c-Jun N-terminal kinase (JNK), extracellular signal-regulated kinase (ERK), and p38 may stimulate Nrf2 phosphorylation [28]. As shown by the IHC results in Figure 6, SP treatment markedly elevated p-Nrf2 expression in the livers of MG-treated rats.

### 2.5. The Potential Mechanism of Scopoletin (SP) on Insulin Resistance

**Figure 7:**
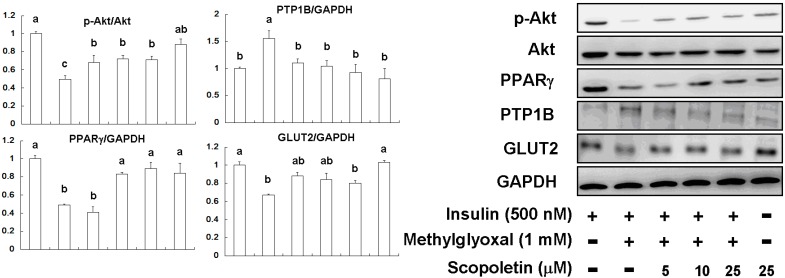
The effects of scopoletin (SP) on Akt phosphorylation, PPARγ, PTP1B, and GLUT2 in MG-induced FL83B hepatocytes. Data were shown as mean ± SD (*n* = 3). (a–c) indicate statistically significant differences *p* < 0.05.

Peroxisome proliferator-activated receptor-gamma (PPARγ) has been reported to activate the phosphatidylinositol 3-kinase (PI3K)/Akt pathway [29]. As shown in Figure 7, insulin resistance was induced in FL83B hepatocytes by MG treatment (1 mM), and thus the p-Akt/Akt ratio was decreased after insulin treatment (500 nM).

**Figure 8:**
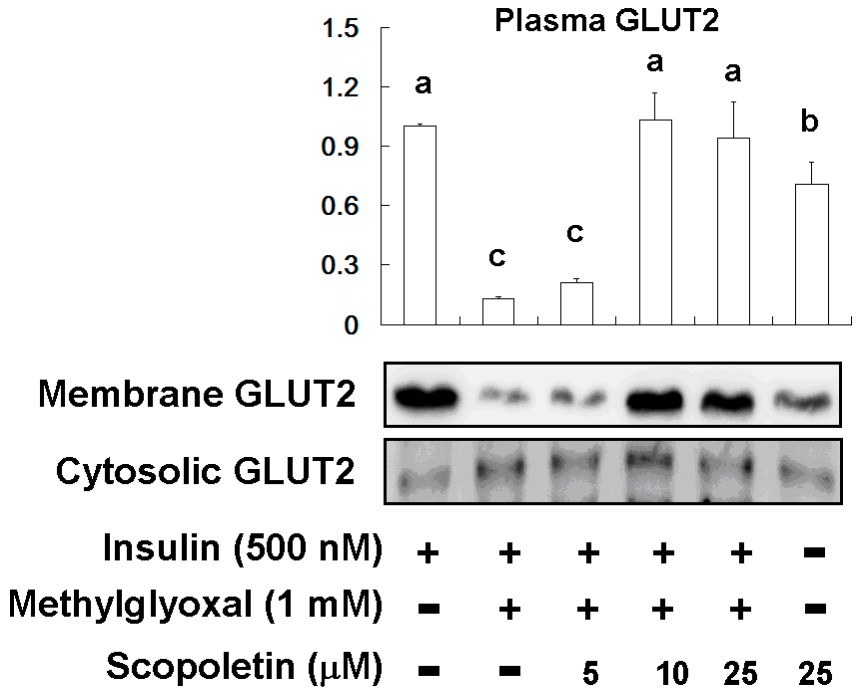
The effects of scopoletin (SP) on plasma translocation of GLUT2 in MG-induced FL83B hepatocytes. Data were shown as mean ± SD (*n* = 3). (a–c) indicate statistically significant differences *p* < 0.05.

SP (5 μM–25 μM) treatment increased Akt phosphorylation, indicating that SP can promote insulin sensitivity. MG induction significantly attenuated PPARγ expression in FL83B hepatocytes; however, SP treatment restored PPARγ expression. MG treatment elevated PTP1B expression and attenuated GLUT2 expression in FL83B hepatocytes. Moreover, SP (10 μM and 25 μM) treatment restored the plasma translocation of GLUT2 in MG-treated FL83B hepatocytes (Figure 8).

### 2.6. Discussion

AGEs can bind covalently to intracellular proteins, lipids, and nucleic acids, thereby affecting their structures and functions [30,31]. MG is detoxified by conversion into d-lactoylglutathione and d-lactate, which is catalyzed in the cytosol of all cells by glyoxalases I and II. This suggests that glyoxalase I completely inhibits the hyperglycemia-induced formation of AGEs; meaning that the role of MG in the formation of AGEs is vital [32]. Additionally, d-lactate quantification in serum and urine was suggested as an effective method to evaluate MG metabolism [33]. Our data reveal that elevated levels of the MG metabolite d-lactate were exhibited among MG-treated rats supplemented with AG or SP when compared to the MG group. Although effective in decreasing the formation of MG, some triosephosphate does escape, and as such cells have developed mechanisms for detoxification. MG can also be metabolized by MG reductase, aldose reductase and 2-oxoaldehyde dehydrogenase to lactaldehyde, hydroxyacetone and pyruvate, respectively. However, the system that handles most of the cellular MG is the glyoxalase system [34]. MG administration lowered glucose tolerance has been reported in rodents [35]. Activation of Nrf2 reduced oxidative damage and insulin resistance *in vitro* and *in vivo* [15,36]. Nrf2 activators can protect against renal damage to improve insulin sensitivity [37]. Nrf2 is a transcription factor for antioxidant enzyme [26,27] and glyoxalase expression [25].

Our results suggest that SP treatment activated hepatic Nrf2 (via Ser40 phosphorylation) (Figure 6), thereby catalyzing MG metabolism to d-lactic acid (Figure 4). In addition, SP also inhibited AGEs generation (Figure 1) in the livers of MG-treated rats (Figure 5). Recently, several studies have reported that SP inhibits AGEs formation [18,38]. Thus, SP is a promising phytochemical therapeutic due to its broad efficacy.

PPARγ is positively associated with the insulin-resistance index, the sensitivity of tissues reacted to insulin is enhanced when the PPARγ agonist treatment [39]. In addition, the PPARγ signaling pathway is a potential target for anti-inflammatory drug development. Previous studies have shown that PPARγ can show anti-inflammatory reaction mediated by PPARγ ligand inhibiting nuclear factor-kappa B (NFκB) [40]. In addition to improving insulin sensitivity, PPARγ agonists are reported to bind to PPAR response element (PPRE) binding sites in GLUT2 promoter regions, thereby up-regulating GLUT2 expression [41].

Recently, research regarding SP has progressed markedly within the field of antidiabetic study. A previous study reported that SP upregulates PPARγ expression, which attenuates high-glucose-induced insulin resistance *in vitro* [42] and *in vivo* [43]. Similar results were found in the current study, in which SP restored PPARγ and GLUT2 expression in MG-treated FL83B hepatocytes (Figure 7).

**Figure 9:**
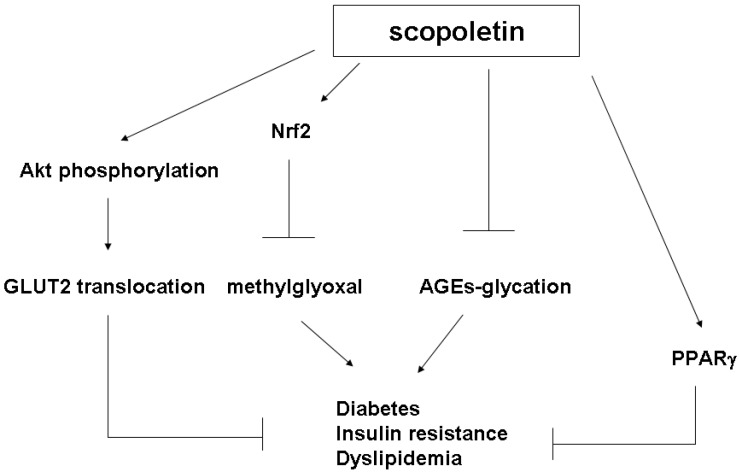
The potential mechanism of scopoletin for diabetes protection.

Akt proteins, a family of docking molecules linking activation of the insulin receptor to essential downstream kinase cascades, are subject to molecular lesions that cause hepatic insulin resistance [39,43]. However, PTP1B attenuates Akt phosphorylation and results in insulin resistance [39,43]. Our results demonstrated that SP treatment suppressed PTP1B expression and activated Akt phosphorylation (Figure 7), and promoted GLUT2 plasma translocation (Figure 8). In our current study, we found that SP restored insulin-stimulated Akt phosphorylation in MG-treated insulin-resistant FL83B hepatocytes and improved OGTT results as well as blood glucose levels in Wistar rats. These effects may mediate the ability of SP to ameliorate insulin resistance, as shown in Figure 9.

Advanced glycation end products (AGEs) are oxidant compounds of pathogenic significance which contributes to the worsening of various chronic illnesses such as diabetes [44]. MG, a major precursor of AGEs and a reactive dicarbonyl metabolite product of glucose metabolism, through reactive oxygen species (ROS) causes tissue injuries [30,45]. Research have suggested that some oxidants such as silymarin, rutin or *N*-acetly cysteine attenuate MG-induced inflammation, tissue damage and symptoms of diabetes [46,47,48,49]. Additionally oxidants such as quercetin, resveratrol and phenolic acid have also been suggested to attenuate oxidative damage through Nrf2 activation [49,50,51]. Furthermore, studies have revealed that dietary phytochemicals such as SP have exhibited anti-oxidative bioactivities [43,52,53]. SP has also been reported to regulate hyperglycemia and diabetic complication [43]; rodent assay also shows the potential to inhibit protein glycation [38]. These various findings suggest that the prospect of effective utilization of antioxidants to attenuate the progression of diabetes is very promising; furthermore this may allow for further studies into the anti-glycation compound of scopoletin.

## 3. Experimental Section

### 3.1. Reagents and Chemicals

Fetal bovine serum (FBS), Aminoguanidine (AG), bovine serum albumin (BSA), F12-K medium, sodium bicarbonate, penicillin, and streptomycin were purchased from HyClone Laboratories (Logan, UT, USA). Glucose, methylglyoxal (MG), and insulin were purchased from Sigma–Aldrich (St. Louis, MO, USA). Anti-AGEs antibodies were purchased from Abcam (Cambridge, MA, USA). Anti-glucose transporter 2 (GLUT2) antibody, anti-protein-tyrosine phosphatase 1B (PTP1B) antibody, and anti-GAPDH antibody were purchased from Santa Cruz Biotechnology (Santa Cruz, CA, USA). Anti-p-nuclear factor-erythroid 2-related factor (Nrf2) antibody was purchased from Bioss (Woburn, MA, USA). Anti-Akt antibody and anti-p-Akt antibody were purchased from Epitomics Inc., (Burlingame, CA, USA). Anti-peroxisome proliferator-activated receptor (PPAR)-γ antibody was purchased from Cayman (Ann Arbor, MI, USA). The Bio-Rad protein assay dye was purchased from Bio-Rad Laboratories (Hercules, CA, USA).

### 3.2. Inhibition of AGEs Formation

AGEs generation was carried out according to the methods of Wang *et al.* [54]. BSA (100 μL, 60 mg/mL), fructose (100 μL, 1.5 M), and sample (100 μL) were mixed and incubated at 50 °C. The fluorescence intensities were determined using a spectrofluorometer (FL × 800, BioTek, Winooski, VT, USA) set at 360 nm (ex) and 460 nm (em).

### 3.3. Animals Treatment

Male Wistar rats (4 weeks of age) were obtained from the National Laboratory Animal Breeding and Research Center (Taipei, Taiwan). Animals were acclimatized for 1 week before use, and were divided at random into four treatment groups (eight rats per group). Animals provided with food and water *ad libitum* and kept under a 12-h light/dark cycle with relative humidity of 60% and a temperature of 25 °C. The protocol complied with guidelines described in the Taiwanese Animal Protection Law, as amended on 17 January 2001 (Hua-Zong-(1)-Yi-Tzi-9000007530, Council of Agriculture, Executive Yuan, Taiwan, ROC). Rats were divided into the following treatment groups and treated for 12 weeks by gavage: (1) control (oral deionized water administration); (2) MG (300 mg/kg, oral administration), (3) MG + scopoletin (SP; 10 mg/kg, oral administration); and (4) MG + aminoguanidine (30 mg/kg, oral administration). Each animal was fed MG first, and then followed by water or chemical. We orally administrated MG and chemicals at 1hr intervals. MG and chemicals were not mixed prior to oral administration in order to emulation high MG concentration in the rats.

### 3.4. Oral Glucose Tolerance Test (OGTT)

The OGTT was performed at week 12 after overnight fasting. Blood samples were collected, and an oral glucose load (2 g·per·kg·bw) was administered. Subsequently, blood glucose levels were determined using a glucose assay kit (BioAssay Systems, Hayward, CA, USA) [55].

### 3.5. Measurements of Serum Biochemical Values

Serum total cholesterol (TC), triacylglycerol (TG), high-density lipoprotein-cholesterol (HDL-C), low-density lipoprotein-cholesterol (LDL-C), and free fatty acid levels were determined by commercial kits from Randox Laboratories Ltd. (Crumlin, Co., Antrim, UK). The D-lactic acid assay kit was purchased from MyBiosource (San Diego, CA, USA). Pro-insulin, the precursor of insulin is cleaved into insulin and C-peptide before the pancreas secretes insulin into the serum. Thus, C-peptide was used to measure insulin secretion from the pancreas.

### 3.6. Immunohistochemistry (IHC) Stain

A frozen section was thaw-mounted onto saline-coated slides, and incubated with 3% H_2_O_2_ for 20 min. After being rinsed twice with PBS, the sections were incubated with skim milk (5%), primary monoclonal antibody, and secondary antibody, respectively. The immunoreactions were visualized by incubation with 3,3'-diaminobenzidine tetrahydrochloride (DAB). The sections were stained with hematoxylin.

### 3.7. Cell Culture

The FL83B mouse hepatocyte cell line was obtained from the Bioresource Collection and Research Center (BCRC) in Taiwan (Hsinchu, Taiwan), and was cultured in F12-K medium supplemented with 10% heat-inactivated FBS and antibiotics (100 units/mL penicillin and 100 μg/mL streptomycin). Cells were cultured at 37 °C in a humidified 5% CO_2_ atmosphere. FL83B cells were treated with MG (1 mM) for 24 h with or without scopoletin, and then insulin was added for 30 min to activate insulin signaling [49].

### 3.8. Membrane and Cytosolic Extraction

Plasma membrane extractions and cytosolic extractions were obtained according to the kit (BioVision, Mountain View, CA, USA).

### 3.9. Western Blot

Proteins separated by sodium dodecyl sulfate-polyacrylamide gel electrophoresis (SDS-PAGE) were electrophoretically transferred to polyvinylidene difluoride (PVDF) membranes. Blots were first incubated with 5% nonfat dry milk, primary monoclonal antibody, and secondary antibody, respectively. Protein expression was detected using an enhanced chemiluminescent (ECL) reagent (Millipore, Billerica, MA, USA).

### 3.10. Statistical Analysis

Data were expressed as mean ± standard deviation (SD). Statistical significance was determined by one-way analysis of variance (ANOVA) using the general linear model procedure of SPSS Version 17.0 (SPSS Inc., Chicago, IL, USA), followed by ANOVA with Duncan’s test. All comparisons were performed relative to control, and significant differences are indicated.

## 4. Conclusions

In summary, the findings of this study reveal that SP could inhibit AGEs production through elevating Nrf2 activity thereby increasing MG metabolism as well as activating insulin signaling (such as PPAR and Akt); finally, SP will attenuate MG-induced impairment of insulin sensitivity. Although further investigation is needed, the beneficial effects of SP on insulin resistance suggest that it may be a useful tool for managing metabolic disorders in humans. Taken together, the results of the current study establish that SP exerts beneficial therapeutic effects against pancreas damage, AGEs accumulation, hyperglycemia, dyslipidemia, and insulin resistance caused by MG administration. These findings suggest that SP may be used as a “functional food” to inhibit the development of diabetes.
